# Supplementary figures and images for: Freshwater Chlorobia Exhibit Metabolic Specialization among Cosmopolitan and Endemic Populations
Source: mSystems. 2021 May 11;6(3):e01196-20. doi: 10.1128/mSystems.01196-20 (PMC8125076; doi:10.1128/mSystems.01196-20)

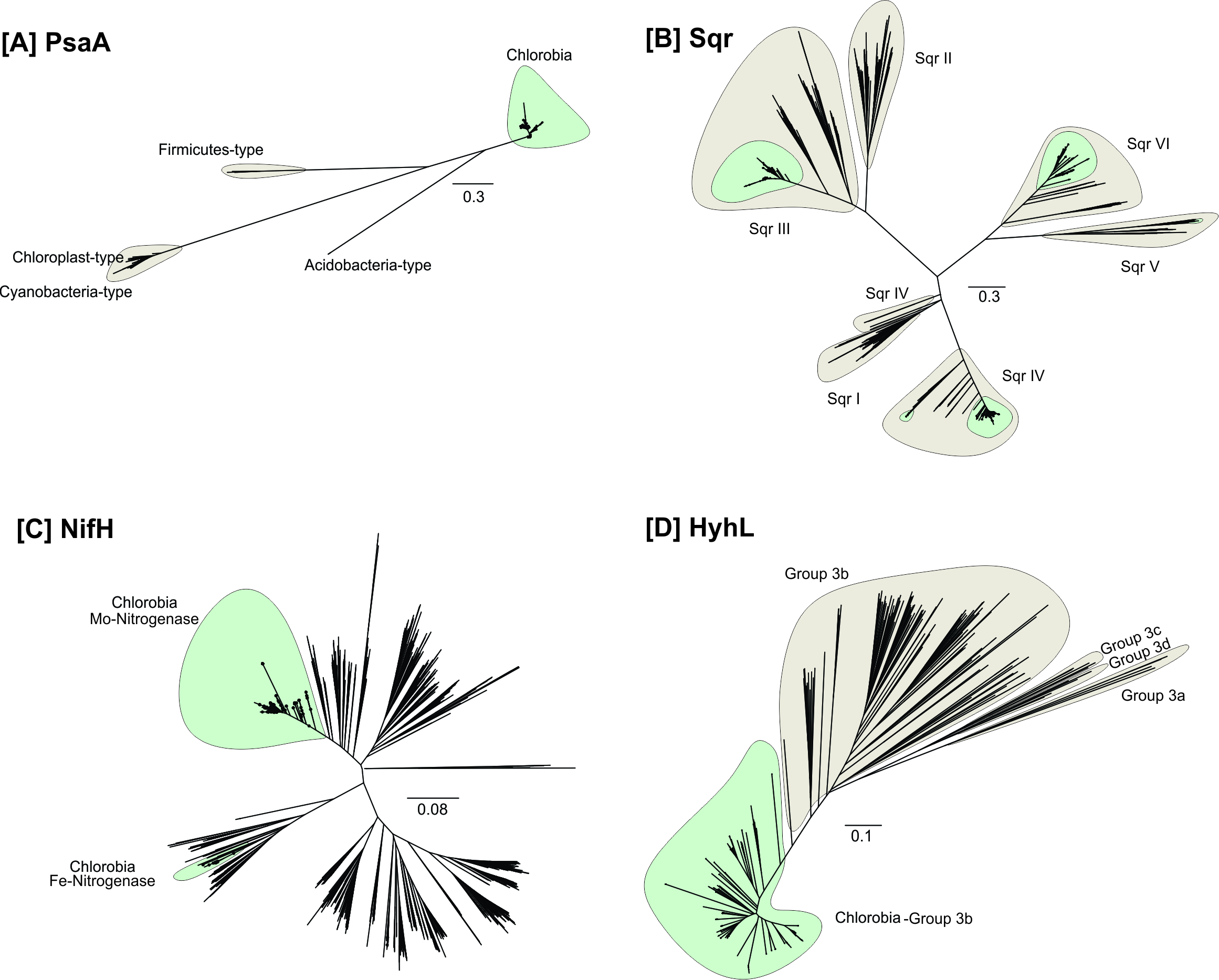

Supplement: FIG S1 [file mSystems.01196-20-sf001.jpg]
